# Targeting c-Jun inhibits fatty acid oxidation to overcome tamoxifen resistance in estrogen receptor-positive breast cancer

**DOI:** 10.1038/s41419-023-06181-5

**Published:** 2023-10-06

**Authors:** Cen Jiang, Youzhi Zhu, Huaying Chen, Junyu Lin, Ruiwang Xie, Weiwei Li, Jiajie Xue, Ling Chen, Xiangjin Chen, Sunwang Xu

**Affiliations:** 1 Central Laboratory, Fujian Medical University Union Hospital, 350001 Fuzhou, China; 2 Department of Thyroid and Breast Surgery, the First Affiliated Hospital, Fujian Medical University, 350005 Fuzhou, China; 3 Department of Thyroid and Breast Surgery, National Regional Medical Center, Binhai Campus of the First Affiliated Hospital, Fujian Medical University, 350212 Fuzhou, China; 4 Fujian Provincial Key Laboratory of Precision Medicine for Cancer, Fuzhou, China

**Keywords:** Cancer metabolism, Breast cancer, Cell death

## Abstract

Tamoxifen-based endocrine therapy remains a major adjuvant therapy for estrogen receptor (ER)-positive breast cancer (BC). However, many patients develop tamoxifen resistance, which results in recurrence and poor prognosis. Herein, we show that fatty acid oxidation (FAO) was activated in tamoxifen-resistant (TamR) ER-positive BC cells by performing bioinformatic and functional studies. We also reveal that CPT1A, the rate-limiting enzyme of FAO, was significantly overexpressed and that its enzymatic activity was enhanced in TamR cells. Mechanistically, the transcription factor c-Jun was activated by JNK kinase-mediated phosphorylation. Activated c-Jun bound to the TRE motif in the CPT1A promoter to drive CPT1A transcription and recruited CBP/P300 to chromatin, catalysing histone H3K27 acetylation to increase chromatin accessibility, which ensured more effective transcription of CPT1A and an increase in the FAO rate, eliminating the cytotoxic effects of tamoxifen in ER-positive BC cells. Pharmacologically, inhibiting CPT1A enzymatic activity with the CPT1 inhibitor etomoxir or blocking c-Jun phosphorylation with a JNK inhibitor restored the tamoxifen sensitivity of TamR cells. Clinically, high levels of phosphorylated c-Jun and CPT1A were observed in ER-positive BC tissues in patients with recurrence after tamoxifen therapy and were associated with poor survival. These results indicate that the assessment and targeting of the JNK/c-Jun-CPT1A-FAO axis will provide promising insights for clinical management, increased tamoxifen responses and improved outcomes for ER-positive BC patients.

## Introduction

Breast cancer (BC) is the most frequently diagnosed cancer and ranks as the second most common cause of cancer-related death in females [1]. BCs are classified according to the expression status of hormone receptors and human epidermal growth factor receptor. Estrogen receptor (ER)-positive BC is the most common BC subtype, accounting for approximately 80% of all BC cases [2]. Endocrine therapy that suppresses estrogen production or targets ER is widely utilised as an adjuvant treatment for patients with ER-positive BC [3]. Tamoxifen is widely used as the standard first-line endocrine therapy, and tamoxifen therapy can reduce cancer recurrence and mortality. However, as many as 40% of patients ultimately develop tamoxifen resistance [4, 5]. Tamoxifen resistance has thus become a major challenge limiting therapy outcomes for ER-positive BC patients.

Both de novo and acquired resistance to tamoxifen occur in patients with BC, and the latter is more commonly used to explain recurrence after long-term tamoxifen therapy in the clinic. In most cases, tamoxifen resistance occurs as a result of genetic or epigenetic alterations in various components of signalling pathways, such as gain-of-function mutations in the ER, altered interactions of the ER with coactivators or corepressors, and compensatory crosstalk between ER and oncogenic signalling pathways [6]. Nonetheless, the molecular mechanism underlying tamoxifen resistance, especially acquired resistance, remains to be further clarified, and this clarification may improve patient responsiveness to clinical treatment.

Tumour cell reprogramming of cellular metabolism supports the molecular interpretation of the malignant biological behaviour of cancer [7]. In addition to glucose and glutamine metabolism, fatty acid metabolism provides large amounts of energy in tumour cells. De novo synthesis of fatty acids supports membrane synthesis for cell proliferation, and fatty acid catabolism mediated via fatty acid oxidation (FAO; also known as β-oxidation) provides more ATP and NADPH than are produced from carbohydrates to enable cell survival [8]. Aberrant activation of FAO is needed for tumour cells to maintain stemness, fuel tumour growth, initiate metastasis, develop drug resistance and evade the immune response [9–13], but the roles of FAO in the tamoxifen resistance of ER-positive BC cells are poorly understood.

FAO is a multistep metabolic process that catalyses the conversion of long-chain fatty acids into acetyl-CoA, which is fully oxidised in the TCA cycle and electron transport chain in mitochondria to produce ATP. Among the multiple catabolic steps of FAO, carnitine palmitoyltransferase 1 (CPT1) is considered the key rate-limiting enzyme [14]. CPT1 overexpression mediated by transcriptional and posttranscriptional mechanisms has been observed in various cancer types [15–18]. Therefore, targeting CPT1 expression or activity has become a therapeutic strategy to restrict the FAO rate during tumour therapy.

In the present study, we found that CPT1A-mediated FAO is enhanced in tamoxifen-resistant ER-positive BC cells; in addition, the expression of CPT1A is increased in ER-positive BC patients with recurrence after tamoxifen therapy compared with those with no recurrence after tamoxifen therapy. We also demonstrated that c-Jun activated by JNK kinase-mediated phosphorylation recruits the CBP/P300 complex to activate CPT1A transcription at the epigenetic level and increases FAO to induce tamoxifen resistance. Finally, we support a drug combination strategy targeting JNK/c-Jun/CPT1A/FAO to overcome tamoxifen resistance in ER-positive BC patients.

## Results

### FAO confers tamoxifen resistance in ER-positive BC cells

**Fig. 1:**
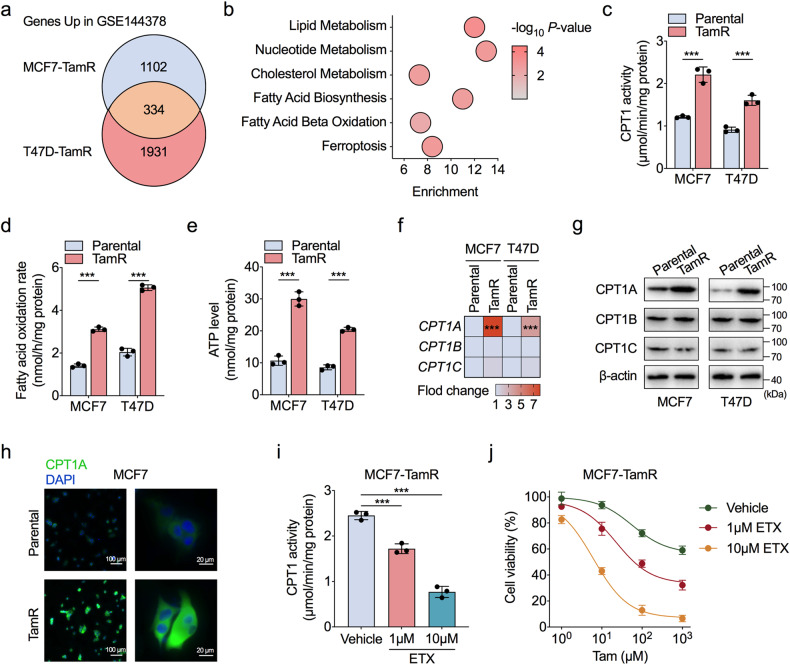
FAO confers tamoxifen resistance in ER-positive BC cells. **a** Venn diagram of overlapping genes significantly upregulated in tamoxifen-resistant (TamR) MCF7 and T47D cells identified in the GEO dataset GSE144378. **b** Gene Ontology analysis of the significantly enriched biological processes for 334 upregulated genes in both MCF7-TamR and T47D-TamR cells. **c**–**e** Comparison of CPT1 enzymatic activities (**c**), fatty acid oxidation (FAO) rates (**d**), and cellular ATP levels (**e**) between TamR and parental MCF7 and T47D cells. **f**, **g** RT-qPCR and western blot assessment of the mRNA (**f**) and protein (**g**) levels of CPT1A, CPT1B, and CPT1C in TamR and parental MCF7 and T47D cells. **h** Representative images for immunofluorescence analysis of CPT1A (green) in TamR and parental MCF7 cells. DAPI (blue) served as a marker for nuclei. **i** Comparison of CPT1 enzymatic activities in MCF7-TamR cells with or without etomoxir (ETX) treatment for 24 h. **j** CCK-8 analysis of MCF7-TamR cells treated with a concentration gradient of tamoxifen combined with or without ETX for 72 h. Unpaired Student’s *t* test in (**c**–**f**, **i**); ***P < 0.001.

To determine the metabolic processes that may be enhanced with the acquisition of tamoxifen resistance in ER-positive BC cells, we analysed the transcriptome data of tamoxifen-resistant (TamR) MCF7 and T47D cells and their parental cells in a published GEO dataset GSE144378 [19]. We found that a total of 996 genes were differentially expressed in both MCF7-TamR and T47D-TamR cells; these included 334 upregulated genes and 632 downregulated genes (Fig. 1a and Supplementary Fig. 1a). Gene Ontology (GO) analysis confirmed that cellular processes, including fatty acid, lipid, nucleotide, and cholesterol metabolism, were significantly enriched in TamR cells (Fig. 1b and Supplementary Fig. 1b).

FAO is a primary source of cell energy, as it yields ATP and cytosolic NADPH, but limited studies have focused on FAO rather than the well-recognised Warburg effect [20]. Recently, FAO has been shown to be a critical inducer of BC stem cell self-renewal and chemoresistance and has been shown to drive BC cell metastasis [10, 21, 22], but its role in tamoxifen resistance in the BC context remains unclear. To clarify the changes in the FAO process in tamoxifen-resistant ER-positive BC cells, we measured FAO activity and ATP production in TamR cells and parental cells. We found that the enzymatic activity of carnitine palmitoyl-transferase 1 (CPT1), the rate-limiting enzyme in FAO, was significantly enhanced in TamR cells (Fig. 1c), and the FAO rate and ATP production rates were accelerated (Fig. 1d, e). Since the expression of CPT1 isoforms exhibits tissue- and cell type-specific patterns [23], we sought to identify the isoform that was altered in TamR cells. The results showed that of the three isoforms of CPT1, only CPT1A was upregulated at both the mRNA and protein levels in TamR cells compared to parental cells (Fig. 1f, g). Immunofluorescence staining of CPT1A in the cytoplasm also indicated increased CPT1A expression in TamR cells (Fig. 1h). In addition, we found that the ectopic expression of CPT1A resulted in a reduction in tamoxifen sensitivity in wild-type ER-positive BC cells (Supplementary Fig. 1c, d). These results indicated that CPT1A-mediated FAO induces tamoxifen resistance.

Etomoxir (ETX), an irreversible CPT1 inhibitor for FAO inhibition, was utilised to investigate the contribution of FAO in inducing tamoxifen resistance in BC cells. ETX treatment decreased the enzymatic activity of CPT1 in an ETX dose-dependent manner and restored tamoxifen sensitivity in two TamR cell lines (Fig. 1i, j and Supplementary Fig. 1e, f), which confirmed that CPT1 activity is required for the development of tamoxifen resistance. Taken together, these data support the finding that CPT1A-mediated FAO activation is a key driver of tamoxifen resistance in ER-positive BC cells.

### c-Jun deletion restores tamoxifen sensitivity

**Fig. 2:**
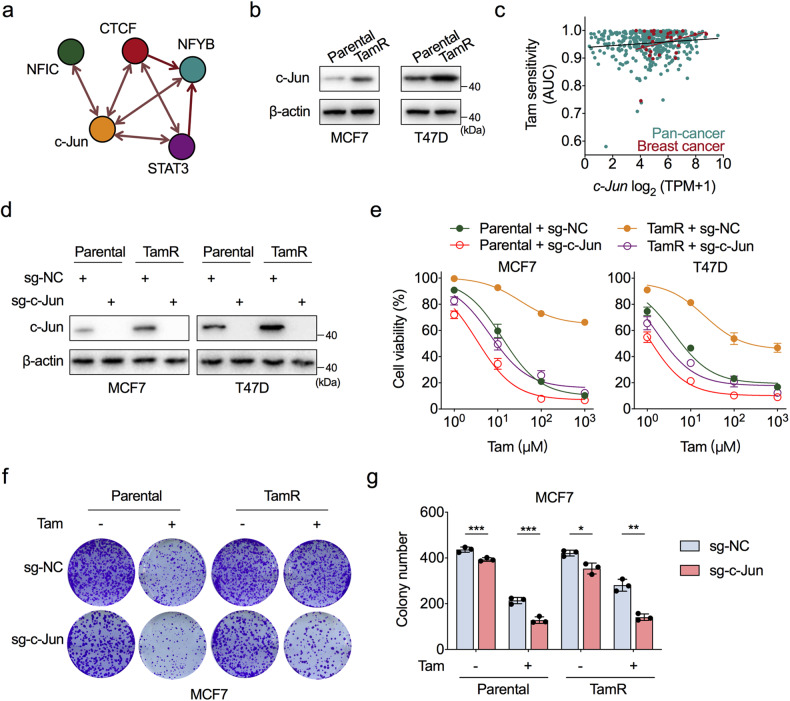
c-Jun deletion restores tamoxifen sensitivity. **a** Top five transcription factors that regulate the expression of upregulated genes in TamR cells, as analysed by the ChEA3 web server. **b** Western blot assessment of c-Jun protein levels in TamR and parental MCF7 and T47D cells. **c** Pearson correlations between c-Jun mRNA levels and tamoxifen sensitivities in pan-cancer and BC cell lines. Pearson *R* = 0.129 for pan-cancer cell lines, and Pearson *R* = 0.286 for BC cell lines. AUC area under curve; a higher AUC indicates stronger cell viability under tamoxifen treatment; TPM transcripts per million. **d** Western blot experiments to validate the c-Jun knockout efficiency in both TamR and parental MCF7 and T47D cells. **e** CCK-8 analysis of TamR and parental MCF7 and T47D cells with or without c-Jun knockout treated with a concentration gradient of tamoxifen for 72 h. **f** Representative images of colony formation assays in TamR and parental MCF7 cells with or without c-Jun knockout treated with tamoxifen (10 μM) or vehicle and stained with crystal violet. **g** Quantification results of the colony formation assays in (**f**). Unpaired Student’s *t* test in (**g**); *P < 0.05, **P < 0.01, ***P < 0.001.

Since CPT1A was significantly upregulated at the transcriptional level in TamR cells, we next explained this finding in terms of transcriptional regulation. To identify the candidate transcription factor (TF) that induces FAO activation in endocrine-resistant BC cells, we performed a TF enrichment analysis for the 334 upregulated genes in TamR cells by using the ChEA3 web server [24]. The results showed that the majority of these upregulated genes was coregulated by the TF c-Jun (Fig. 2a). We confirmed that c-Jun expression was markedly increased in TamR cells compared to parental cells (Fig. 2b).

c-Jun is a component of the AP-1 transcription factor family that regulates target gene transcription to deregulate cancer-relevant signalling pathways. The oncogenic role of c-Jun in the malignant phenotype of BC has been recognised [25]. Next, we analysed the biological connection between c-Jun and tamoxifen resistance in ER-positive BC cells. The corresponding association between gene expression and drug sensitivity obtained from the Cancer Dependency Map (https://depmap.org/portal/) showed that higher c-Jun expression was correlated with greater tamoxifen resistance in both pancancer and BC cell lines (Fig. 2c).

To directly determine the association between c-Jun expression and tamoxifen resistance in ER-positive BC cells, we deleted endogenous c-Jun expression in both TamR and parental cells via the CRISPR/Cas9 approach (Fig. 2d). The cell viability assay and colony formation assay confirmed that knockout of c-Jun enhanced the tamoxifen mediated cytotoxic effect in parental cells and also remarkably restored tamoxifen sensitivity in TamR cells, which reached the level similar to that in parental cells (Fig. 2e–g and Supplementary Fig. 2a, b). In addition, we found that c-Jun deletion slightly decreased the proliferative ability of ER-positive BC cells (Fig. 2f, g). Taken together, these results demonstrate that c-Jun is a key oncogene in the induction of tamoxifen resistance and tumour growth arrest in ER-positive BC.

### c-Jun activates FAO to induce tamoxifen resistance

**Fig. 3:**
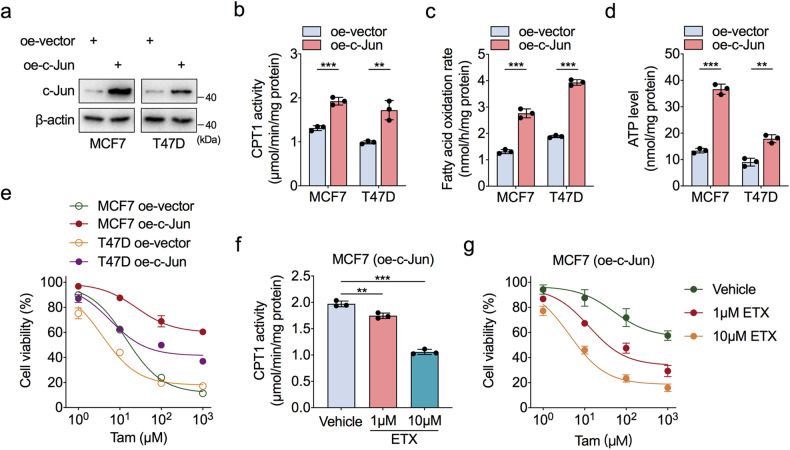
c-Jun activates FAO to induce tamoxifen resistance. **a** Western blot analysis to detect the c-Jun overexpression in both wild-type MCF7 and T47D cells. **b**–**d** Comparison of CPT1 enzymatic activities (**b**), FAO rates (**c**), and cellular ATP levels (**d**) in wild-type MCF7 and T47D cells transfected with c-Jun overexpressing construct or empty vector. **e** CCK-8 analysis of c-Jun or empty vector transfected wild-type MCF7 and T47D cells which were treated with a concentration gradient of tamoxifen for 72 h. **f** CPT1 enzymatic activities in c-Jun-overexpressing wild-type MCF7 cells with or without ETX treatment for 24 h. **g** CCK-8 analysis of c-Jun-overexpressing wild-type MCF7 cells treated with a concentration gradient of tamoxifen combined with or without ETX for 72 h. Unpaired Student’s *t* test in (**b**–**d**, **f**); **P < 0.01, ***P < 0.001.

To confirm the link between c-Jun and FAO in the tamoxifen resistance of ER-positive BC cells, we overexpressed c-Jun in wild-type MCF7 and T47D cells (Fig. 3a). We found that ectopically expressed c-Jun enhanced CPT1 enzymatic activity, accelerated the FAO rate, and promoted ATP production (Fig. 3b–d). Moreover, c-Jun overexpression abrogated the tamoxifen-mediated growth-inhibitory effects in ER-positive BC cells (Fig. 3e), which suggested that c-Jun contributes to tamoxifen resistance by enhancing FAO activity.

Then, we treated c-Jun-overexpressing cells with the FAO inhibitor ETX to determine whether c-Jun-mediated activation of FAO was required for tamoxifen resistance. The results showed that ETX treatment blocked the c-Jun-mediated increase in CPT1 activity and reversed the enhancement of tamoxifen resistance mediated by c-Jun (Fig. 3f, g and Supplementary Fig. 3a, b). Together, these data provide the biological connection between c-Jun and FAO and confirm that FAO is critical for c-Jun-induced tamoxifen resistance in ER-positive BC cells.

### c-Jun recruits CBP/P300 to activate CPT1A transcription

**Fig. 4:**
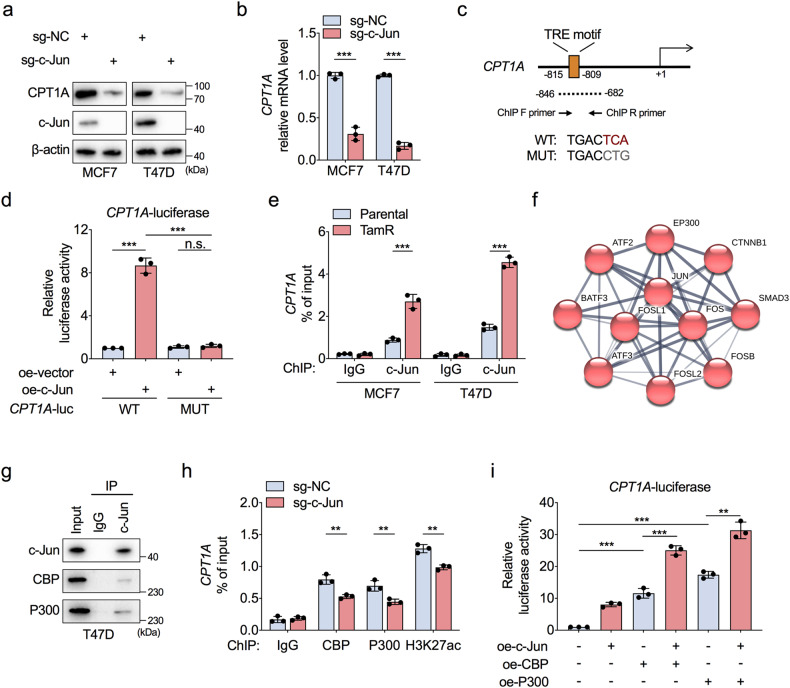
c-Jun recruits the CBP/P300 to activate CPT1A transcription. **a**, **b** Western blot and RT-qPCR assessment of the protein (**a**) and mRNA (**b**) levels of CPT1A in wild-type MCF7 and T47D cells with or without c-Jun knockout. **c** Schematic diagram of the TRE motif location on the wild-type (WT) or mutant (MUT) promoter of CPT1A for luciferase reporter assays. TRE, TPA-responsive element. **d** HEK293T cells were transfected with empty vector or c-Jun-overexpressing plasmid to analyse the luciferase reporter activity driven by the WT or MUT CPT1A promoter. **e** Chromatin immunoprecipitation quantitative PCR (ChIP-qPCR) analysis of c-Jun occupancy on the CPT1A promoter region around the TRE motif in TamR and parental MCF7 and T47D cells. **f** STRING analysis of proteins interact with c-Jun. **g** Immunoprecipitation assay to confirm the interaction between c-Jun and CBP/P300 in T47D cells. **h** ChIP-qPCR assessment for the comparison of CBP, P300, and H3K27ac occupancy on the CPT1A promoter in MCF7 cells with or without c-Jun knockout. **i** HEK293T cells were transfected with empty vector, c-Jun-, CBP-, or P300-overexpressing plasmid alone or together as indicated to analyse the luciferase reporter activity driven by the WT CPT1A promoter. Unpaired Student’s *t* test in (**b**, **e**, **h**), and paired Student’s *t* test in (**d**, **i**); **P < 0.01, ***P < 0.001, n.s., not significant.

c-Jun can form homodimers or heterodimers with other AP-1 members to bind the cAMP-responsive element (CRE) or TPA-responsive element (TRE) and thus regulate the transcription of specific genes [26]. As predicted via use of the JASPAR database, a TRE motif (5′-TGACTCA-3′) at −815 to −809 bp upstream of the transcription start site (TSS) in the CPT1A promoter was identified (Supplementary Fig. 4a), which suggested that c-Jun might directly bind to the CPT1A promoter and activate CPT1A transcription to enhance FAO. As validated by protein and mRNA studies, c-Jun deletion inhibited CPT1A expression at the transcriptional level (Fig. 4a, b).

To confirm the binding ability of c-Jun to the TRE motif in the CPT1A promoter, we generated luciferase reporter constructs containing the wild-type TRE motif or a mutant form to which c-Jun could not bind and then performed luciferase reporter assays (Fig. 4c). The results showed that the wild-type CPT1A promoter induced a substantial increase in luciferase activity with ectopic expression of c-Jun, while the mutant form completely inhibited the increase in activity induced by c-Jun overexpression (Fig. 4d). In addition, chromatin immunoprecipitation quantitative PCR (ChIP-qPCR) assays confirmed that the occupancy of c-Jun on the CPT1A promoter was significantly increased in TamR cells (Fig. 4e). These data suggest that the increase in CPT1A expression is mediated by the direct binding of c-Jun to the TRE motif within the promoter to drive CPT1A transcription.

TFs usually collaborate with epigenetic modifications to activate or repress target gene transcription. According to a STRING database analysis, c-Jun functionally interacted with P300 (Fig. 4f); an immunoprecipitation assay showed that c-Jun interacted with CBP/P300 to form a complex in ER-positive BC cells (Fig. 4g). CBP/P300 is a histone acetyltransferase that catalyses the acetylation of histone 3 lysine 27 (H3K27ac), which is a hallmark of active transcription [27]. Functionally, c-Jun deletion prevented CBP/P300 binding to the CPT1A promoter, which was followed by a reduction in the H3K27ac level around the TRE motif within the CPT1A promoter (Fig. 4h). Consistently, CBP/P300 synergised with c-Jun to enhance the transcriptional activity of the CPT1A promoter, but this effect was diminished when c-Jun was deleted (Fig. 4i and Supplementary Fig. 4b). These results confirmed that c-Jun is required for CBP/P300 binding to the CPT1A promoter and that c-Jun collaborates with H3K27ac mark-related signalling to prime CPT1A transcription.

### JNK-dependent c-Jun phosphorylation activates FAO

**Fig. 5:**
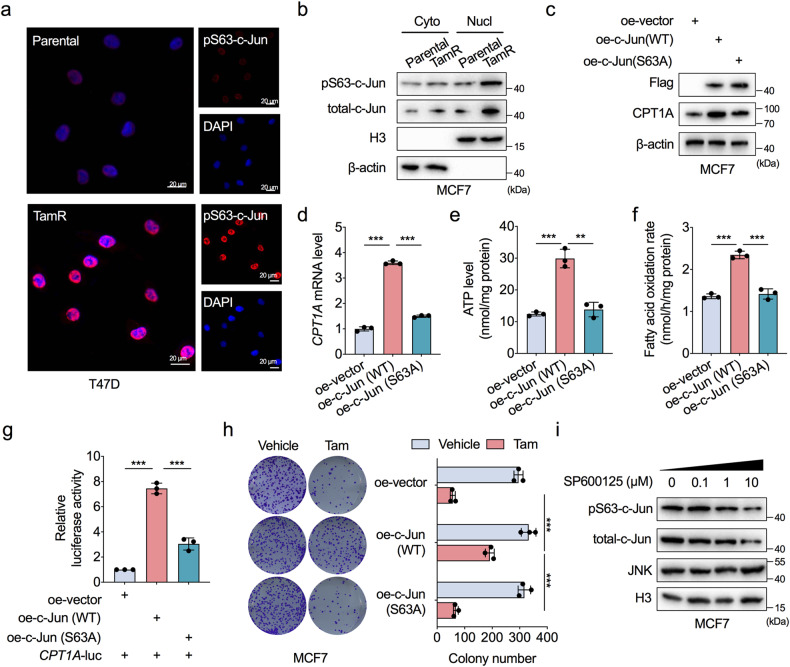
JNK-dependent c-Jun phosphorylation activates FAO. **a** Representative images for immunofluorescence analysis of phosphorylated c-Jun at the Ser63 site (pS63-c-Jun, red) in TamR and parental T47D cells. DAPI (blue) served as a marker for nuclei. **b** Western blot analysis showing the different levels of pS63-c-Jun in the cytoplasm and nuclei between TamR and parental MCF7 cells. **c**–**f** Comparison of CPT1A protein levels (**c**), CPT1A mRNA levels (**d**), cellular ATP levels (**e**), and FAO rates (**f**) in MCF7 cells transfected with empty vector, wild-type c-Jun construct (WT), or Ser63 phosphorylation disabled mutant form of c-Jun construct (S63A). **g** HEK293T cells were transfected with CPT1A luciferase reporter plasmids together with empty vector, WT c-Jun, or S63A c-Jun construct to analyse the luciferase reporter activity of the CPT1A promoter. **h** Representative images (left) and quantification results (right) of the colony formation assays for MCF7 cells transfected with empty vector, WT c-Jun, or S63A construct and treated with tamoxifen (10 μM) or vehicle. **i** Western blot assessment of the protein levels of pS63-c-Jun, total c-Jun, and JNK in nuclei of MCF7 cells treated with a concentration gradient of the JNK inhibitor SP600125 for 24 h. Unpaired Student’s *t* test in (**d**–**f**, **h**), and paired Student’s *t* test in (**g**); **P < 0.01, ***P < 0.001.

Phosphorylation is required for the transactivation activity of c-Jun. In particular, the JNK family of MAP kinase phosphorylates c-Jun at Ser-63, resulting in translocation of c-Jun to the nucleus and the binding of c-Jun to chromatin for induction of target gene transcription [28]. Moreover, we found that activated JNK cascade signalling was significantly enriched in TamR cells (Supplementary Fig. 5a). Therefore, we next investigated the possible role of c-Jun phosphorylation in the tamoxifen resistance of ER-positive BC cells. Phosphorylated c-Jun at Ser-63 was significantly enriched in the nucleus of TamR cells (Fig. 5a, b and Supplementary Fig. 5b). Therefore, we generated a loss-of-function c-Jun S63A construct that cannot be phosphorylated and transfected it into MCF7 and T47D cells (Fig. 5c and Supplementary Fig. 5c). In contrast to the c-Jun wild-type construct, the c-Jun S63A mutant form failed to promote CPT1A expression at either the mRNA or protein level (Fig. 5c, d and Supplementary Fig. 5c, d); furthermore, the c-Jun S63A mutant failed to increase ATP production and the FAO rate in ER-positive BC cells (Fig. 5e, f and Supplementary Fig. 5e, f). In addition, the c-Jun S63A construct induced only minor effects on the promoter activity of CPT1A compared to profound effects mediated by the c-Jun wild-type construct (Fig. 5g). Moreover, wild-type c-Jun induced tamoxifen resistance in MCF7 cells, but the c-Jun S63A mutant exerted little effect on tamoxifen sensitivity in MCF7 cells (Fig. 5h).

In addition, with the application of SP600125, a pan JNK inhibitor (JNKi), the protein levels of c-Jun and Ser-63-phosphorylated c-Jun in the nucleus were markedly decreased in a dose-dependent manner in MCF7 cells, suggesting that JNK activity is needed for c-Jun activation in ER-positive BC cells (Fig. 5i). These results support the finding that JNK-dependent c-Jun phosphorylation activates FAO and tamoxifen resistance in ER-positive BC cells.

### Blocking JNK/c-Jun increases tamoxifen sensitivity via FAO inhibition

**Fig. 6:**
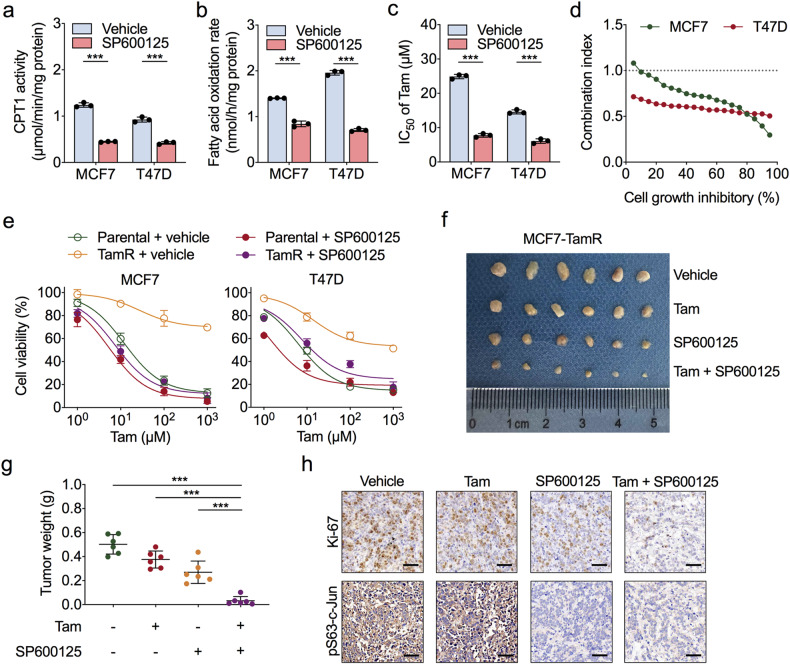
JNK inhibitors sensitise ER-positive BC cells to tamoxifen by inhibiting c-Jun-induced FAO. **a**, **b** Comparison of CPT1 enzymatic activities (**a**) and FAO rates (**b**) in MCF7 and T47D cells treated with the JNK inhibitor SP600125 (10 μM) or vehicle for 24 h. **c** Cell viability analysis for MCF7 and T47D cells treated with a concentration gradient of tamoxifen combined with SP600125 (10 μM) or vehicle for 72 h. IC50, half maximal inhibitory concentration. **d** Combination index-fraction affected plots of combined treatment of tamoxifen and SP600125 in MCF7 and T47D cells. Plots were generated using CompuSyn software. Combination index (CI) < 1, CI = 1, and CI > 1 indicate synergism, additive effect, and antagonism, respectively. A smaller CI value indicates stronger synergism. **e** CCK-8 analysis for TamR and parental MCF7 and T47D cells treated with a concentration gradient of tamoxifen combined with SP600125 (10 μM) treatment or not for 72 h. **f** Representative data of tumours in nude mice bearing MCF7-TamR cells received different treatment, *n* = 6/group. **g** Statistical analysis of mouse tumour weight in different groups, *n* = 6/group. **h** Representative immunohistochemistry (IHC) staining for Ki-67 (upper) and pS63-c-Jun (lower) in formalin-fixed tumour sections from the indicated treatment groups. Scale bars = 50 μm. Unpaired Student’s *t* test in (**a**–**c**, **g**); ***P < 0.001.

Since JNKi inhibits the phosphorylation of c-Jun, we next evaluated whether JNKi attenuates FAO in ER-positive BC cells. Compared to vehicle treatment, treatment with SP600125 significantly reduced the CPT1 activity and FAO rate in MCF7 and T47D cells (Fig. 6a, b), which demonstrated that JNK-mediated c-Jun phosphorylation modulated the activity of FAO in ER-positive BC cells. Thus, we examined the potential synergistic effect of a JNKi and tamoxifen. As expected, combination treatment of SP600125 and tamoxifen sensitised MCF7 and T47D cells to the effects of tamoxifen (Fig. 6c). A Chou–Talalay assay confirmed the synergistic effects of tamoxifen and SP600125 in MCF7 and T47D cells (Fig. 6d). Moreover, SP600125 treatment decreased TamR cell viability in vitro (Fig. 6e).

To further determine the efficacy of SP600125 in the growth arrest in TamR cell tumours in vivo, we subcutaneously implanted MCF7 TamR cells into athymic nude mice. There was little significant reduction in tumour weight in the TamR tumour-bearing group that underwent tamoxifen treatment, but SP600125 treatment diminished tumour growth and inhibited the phosphorylation of c-Jun in vivo (Fig. 6f–h). The combination of tamoxifen and SP600125 significantly diminished tumour weight, tumour cell proliferation and c-Jun phosphorylation (Fig. 6f–h and Supplementary Fig. 6a) without inducing nephrotoxicity or liver toxicity (Supplementary Fig. 6b). Taken together, these data suggest that targeting JNK/c-Jun resensitises tamoxifen-resistant BC cells to tamoxifen therapy and abrogates tumour growth.

### c-Jun predicts tamoxifen therapy outcomes in breast cancer patients

**Fig. 7:**
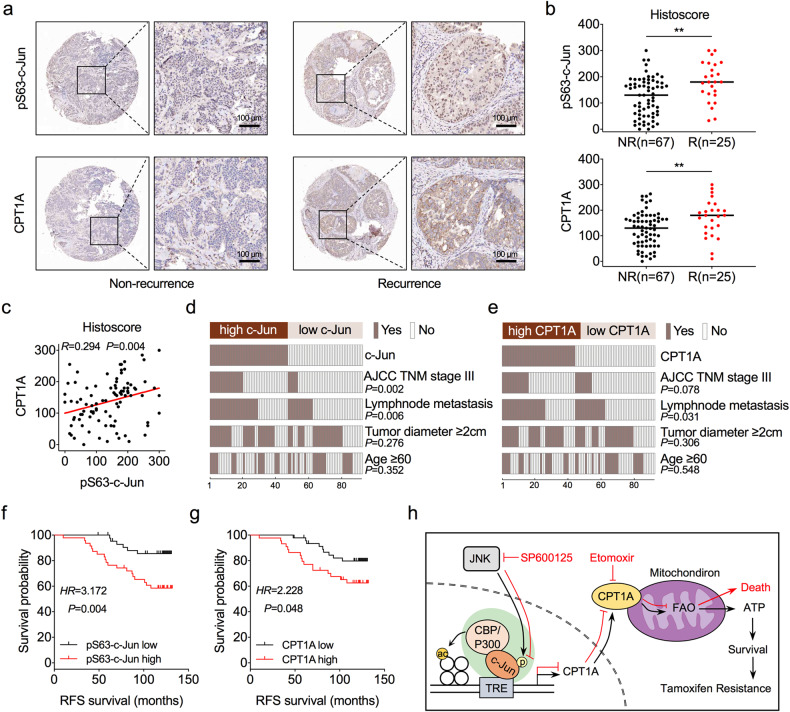
c-Jun predicts tamoxifen therapy outcomes in ER-positive BC patients. **a** Representative IHC staining of pS63-c-Jun (upper) and CPT1A (lower) proteins in tumour tissues from ER-positive BC patients received tamoxifen therapy with or without recurrence after. **b** Statistical analysis of the histoscore of pS63-c-Jun (upper) and CPT1A (lower) proteins in tumour tissues from ER-positive BC patients received tamoxifen therapy with or without recurrence. NR, non-recurrence; R, recurrence. Unpaired Student’s *t* test; **P < 0.01. **c** Correlations between pS63-c-Jun and CPT1A protein levels in ER-positive BC tissues. Pearson correlation coefficient for statistical analysis. **d** The heatmap illustrates the association of different clinical characteristics in ER-positive BC patients with high and low expression of pS63-c-Jun. **e** The heatmap illustrates the association of different clinical characteristics in ER-positive BC patients with high and low expression of CPT1A. Statistical significance was assessed by the Chi-square test in (**d**, **e**). **f** Recurrence-free survival (RFS) was compared between patients with high and low expression of pS63-c-Jun. **g** RFS was compared between patients with high and low expression of CPT1A. Log-rank test in (**f**, **g**); HR, Hazard ratio. **h** Model depicting the role of the JNK/c-Jun-CPT1A-FAO axis in driving tamoxifen resistance in ER-positive BC.

To further assess the relevance of c-Jun phosphorylation and CPT1A expression on the clinical outcomes of ER-positive BC patients, we assessed and quantified the expression of Ser-63-phosphorylated c-Jun and CPT1A by immunohistochemistry in ER-positive BC tissues. We found that high expression of pS63-c-Jun and CPT1A was more likely to be detected in patients with recurrence after tamoxifen therapy than in patients without recurrence (Fig. 7a, b). In addition, the expression level of pS63-c-Jun was positively correlated with CPT1A expression in ER-positive BC tissues (Fig. 7c and Supplementary Fig. 7a).

We next evaluated the relationship between the expression levels of pS63-c-Jun and CPT1A and the different clinicopathological features in patients with ER-positive BC. High pS63-c-Jun expression was correlated with advanced American Joint Committee on Cancer (AJCC) tumour node metastasis (TNM) stage and lymph node metastasis (Fig. 7d), and high CPT1A was also associated with lymph node metastasis (Fig. 7e). Finally, high levels of pS63-c-Jun and CPT1A were closely associated with shorter recurrence-free survival (RFS) and overall survival (OS) (Fig. 7f, g and Supplementary Fig. 7b, c). In addition, patients with high levels of both pS63-c-Jun and CPT1A showed a worsened prognosis after tamoxifen therapy than those with low expression of either one or both proteins (Supplementary Fig. 7d, e). These data indicate that phosphorylated c-Jun and CPT1A are pathologically and clinically associated with cancer recurrence and survival outcomes in ER-positive BC patients who received tamoxifen therapy.

## Discussion

Endocrine therapy, particularly tamoxifen therapy, has been widely used as adjuvant treatment for patients with ER-positive BC after surgery, especially in premenopausal women. The initial responses to surgery and tamoxifen therapy are often good in ER-positive BC patients, but recurrence of tamoxifen-resistant cancer usually occurs, and these tumours progress into advanced and metastatic stages, leading to poor survival outcomes. Thus, discovering the molecular mechanisms underlying tamoxifen resistance in ER-positive BC cells is essential for finding novel therapeutic strategies to improve patient outcomes. Here, we show that c-Jun and CPT1A activities were closely associated with tamoxifen resistance and correlated with poor survival outcomes for ER-positive BC patients who received tamoxifen therapy. Mechanistically, JNK specifically phosphorylated c-Jun to increase the transcriptional activity of c-Jun, which recruited CBP/P300 to drive the transcription of CPT1A at the epigenetic level, followed by enhancement of FAO to induce tamoxifen resistance in ER-positive BC cells (Fig. 7h).

Genetic and epigenetic alterations that lead to tamoxifen or endocrine resistance have been widely reported [29–33]. Recent studies have shown that metabolic reprogramming provides survival advantages protecting tumour cells from endocrine therapy-induced death and in turn confers endocrine resistance [34, 35]. Previous studies have provided evidence showing that FAO induces paclitaxel resistance [10, 21], trastuzumab resistance [15], endoxifen resistance [36], and even radioresistance [37] in BC cells. Moreover, FAO can protect tumour cells from chemotherapy-induced apoptosis by maintaining mitochondrial integrity [22]. However, the potential driving and therapeutic role of FAO in tamoxifen resistance in ER-positive BC is poorly understood. Through a combination of transcriptomic, cytobiological, biochemical, pharmacological, and clinical experiments, we demonstrated that the FAO process is enriched in tamoxifen-resistant ER-positive BC cells with the increased enzymatic activity of CPT1A and that the CPT1A-FAO axis promotes the development of tamoxifen resistance in ER-positive BC cells.

CPT1 anchors to the mitochondrial outer membrane and facilitates the entry of long-chain fatty acids into mitochondria by loading fatty acyl groups onto carnitine to support FAO. Three subtypes of CPT1 family proteins, including liver type (CPT1A), muscle type (CPT1B) and brain type (CPT1C), can be expressed in almost all tissues and cell types but exhibit tissue and cell specificity, and even the kinetics of CPT1 subtypes exhibit unequal dominance [23]. We confirmed that all three CPT1 subtypes were expressed in ER-positive BC cells, but the CPT1A subtype was expressed at higher levels in tamoxifen-resistant cells. This evidence suggests that the CPT1A subtype, but not the other two subtypes, drives FAO activity in tamoxifen-resistant cells. Integrative genomic analysis revealed that the CPT1A DNA copy number is amplified and that CPT1A is essential to the viability of ER-positive BC cells [38]. Moreover, the overexpressed CPT1A is critical for enhancing FAO to promote proliferation in ER-positive BC cells, suggesting an oncogenic role of CPT1A-mediated FAO in the BC context [39]. In the current study, we provide evidence showing that CPT1A is a key driver that primes tamoxifen resistance development.

Given that CPT1A-enhanced FAO can promote the acquisition of malignant phenotypes such as cell proliferation and tamoxifen resistance in ER-positive BC cells, CPT1A-FAO might be an effective target for overcoming tamoxifen resistance in BC cells. Recent reports have indicated that pharmacologic inhibition of CPT1A and FAO could prevent ER-positive BC tumour growth and cell proliferation [10, 15, 39, 40]. In the present study, we demonstrated that inhibition of CPT1A-mediated FAO with a CPT1 inhibitor re-sensitised ER-positive BC cells to tamoxifen therapy, which broadened the therapeutic effects of CPT1A/FAO inhibitors in tumours. Although etomoxir has been used in the clinic for the treatment of heart failure [41, 42], the safety and efficiency of CPT1 inhibitors combined with endocrine therapy in the clinic needs to be validated.

Here, we also show that CPT1A is more highly expressed in BC patients with relapse, who have worsened survival outcomes in the clinic, suggesting that CPT1A might be a prognostic indicator for ER-positive BC. Although the three subtypes of CPT1 share highly similar protein sequences, they are located on different chromosomes, and their expression is regulated by different biological mechanisms. Enhanced CPT1A expression partially results from the amplification of chromosome 11q13-14 in ER-positive BC, but the copy number gains of CPT1A do not occur in all BC with increased CPT1A expression [38, 39]. The expression of CPT1A is also activated by epigenetic control mechanisms, including various transcription factors or transcription coactivators, in different cancer types [43–46]. However, the regulatory factor that primes CPT1A activation in ER-positive BC cells is unclear. Through bioinformatic prediction and biochemical assays, we identified that the transcription factor c-Jun activates the transcription of CPT1A via direct and indirect mechanisms. On the one hand, c-Jun binds to the TRE motif within the promoter of CPT1A to directly activate CPT1A transcription. On the other hand, c-Jun recruits CBP/P300 to catalyse the acetylation of H3K27 near the promoter of CPT1A to increase chromatin accessibility, which is essential for transcription initiation. These findings indicate that c-Jun promotes the expression of CPT1A to increase the FAO rate during the acquisition of the tamoxifen resistance phenotype. In addition to epigenetic control, the crosstalk between factors involved in different metabolic processes can regulate CPT1A activity. A recent study revealed that a mitochondrial flavoprotein promoted CPT1A transcription levels by modulating mitochondrial function [40]. This finding inspired us to conclude that cellular metabolic processes might also exert a regulatory effect on CPT1A expression in ER-positive BC cells. Collectively, the expression of CPT1A in ER-positive BC cells might be controlled by multiple mechanisms, including genetic alteration, epigenetic regulation, and/or cellular metabolic signalling. Our work extends the current knowledge on how c-Jun-mediated transcriptional activation regulates the tamoxifen therapeutic response in patients with ER-positive BC.

c-Jun promotes BC cell growth by interacting with ER to reprogramme ER chromatin-binding to cell growth-related genes [25, 47]. Here, we found that c-Jun deletion impaired the growth of ER-positive BC cells, suggesting that c-Jun serves as a therapeutic target for ER-positive BC. Because direct inhibition of transcription factors is pharmacologically challenging, we targeted the kinases upstream of c-Jun, which might be an effective therapeutic strategy to inhibit the transcriptional activity of c-Jun. c-Jun is specifically phosphorylated by JNK kinase in BC cells [48], and JNK has become a potential target for anticancer therapy. Interestingly, increased mitochondrial FAO activates the JNK pathway in MCF7 cells [49]. In conjunction with our findings, these findings collectively raise the possibility that CPT1A/FAO might form a feedback loop induced by JNK activity to generate tamoxifen resistance in ER-positive BC cells. A number of ATP-competitive and ATP-noncompetitive JNK inhibitors have been developed, and these inhibitors have induced good therapeutic responses in cell-based experiments and animal models of cancer, but their clinical use has not been reported [50]. JNK inhibitors also induce cell cycle arrest in BC cells by inhibiting the kinase activity of JNKs [51]. SP600125, the most studied ATP-competitive JNK inhibitor, showed remarkable anticancer effects to inhibit the spherogenesis of MCF7 cells [49] or overcome multiple-drug resistance in cancers when applied in combination with traditional chemotherapeutic agents [52], but its role in sensitising cells to tamoxifen therapy is unknown. We report that SP600125 inhibited FAO, synergistically enhanced the cytotoxic effect of tamoxifen and reversed tamoxifen resistance in ER-positive BC cells, providing a new idea explaining how JNK inhibitors exert anticancer effects. However, several studies reported that SP600125 also showed anticancer effects in a JNK-independent manner, and these off-target effects of SP600125 might compromise the inhibitory effect of SP600125 on cell proliferation [53, 54]. In addition, due to the lack of specificity of SP600125, which indiscriminately inhibits the phosphorylation of all JNK substrates or other off-target phosphorylation substrates, and the high therapeutic dose [55], the clinical translation value of SP600125 is limited. Therefore, it is necessary to further search for a highly specific inhibitor that blocks JNK phosphorylation of c-Jun to minimise the off-target effects and provide more precise anticancer effects.

The JNK signalling pathway is required for the tumorigenesis and metastasis of BC and is significantly activated in advanced and metastatic BC [48, 56], which could explain why phosphorylated c-Jun was enriched in advanced AJCC TNM stages of ER-positive BC in our work. In addition, excessive JNK activity induces chemoresistance in BC [57] and primes acquired resistance to tamoxifen [58, 59] and aromatase inhibitors [60]. In our acquired tamoxifen-resistant cell model generated in vitro, we confirmed that JNK-activated c-Jun contributed to the acquired resistance to tamoxifen in ER-positive BC. Both de novo and acquired resistance are important for the generation of tamoxifen resistance in ER-positive BC. Among these two approaches, acquired resistance is the most frequent type of tamoxifen resistance in advanced BC contexts [61], but genetic alterations and oncogenic signalling activation lead to the generation of de novo resistance in advanced BC [62]. Here, we found that ER-positive BC tissues with advanced stage showed higher expression of phosphorylated c-Jun. It is reasonable to ask whether activated JNK/c-Jun primes ER-positive BC in advanced stages to induce de novo resistance to tamoxifen; this is possibility that needs to be further explored.

In summary, we discovered a novel biological mechanism by which the JNK/c-Jun–CPT1A–FAO axis induces tamoxifen resistance in ER-positive BC cells. Our work suggests a potentially effective therapeutic strategy to overcome tamoxifen resistance and restore tamoxifen sensitivity by targeting JNK/c-Jun and CPT1A-FAO. This strategy may lead to promising treatment options that improve the clinical outcomes of ER-positive BC patients.

## Materials and methods

### Clinical specimens

A total of 92 cases of ER-positive BC tissues with complete clinicopathological and follow-up data were retrospectively obtained from BC patients received radical mastectomy in Department of Thyroid and Breast Surgery, The First Affiliated Hospital of Fujian Medical University between January 2008 and December 2015 with the inclusion criteria of (1) definitive BC diagnosis with ER-positive status by pathology, (2) no radiotherapy, neoadjuvant chemotherapy or targeted therapy before surgery, (3) received standard tamoxifen therapy at least five years or at the time of relapse. The Ethics Committees of The First Affiliated Hospital of Fujian Medical University approved the study protocols. Written informed consents were obtained from all participants in this study. All the research was carried out in accordance with the provisions of the Helsinki Declaration of 1975.

### Cell culture and reagents

Human ER-positive breast cancer cell lines MCF7 and T47D (ATCC), and HEK293T (ATCC) cells were cultured in DMEM (Hyclone) with 10% fetal bovine serum (Gibco) and 1% penicillin–streptomycin (Hyclone) at 37 °C in a humidified 5% CO_2_ atmosphere. Tamoxifen-resistant (TamR) MCF7 and T47D cells were generated as described previously [63]. In brief, cells were cultured in the presence of increasing concentrations of 4-hydroxytamoxifen (Sigma-Aldrich) starting at 0.5 μM, and finally gradually increased up to 5 μM 4-hydroxytamoxifen when the growth of the cells could not be inhibited in this concentration. In parallel, parental MCF7 and T47D cells were cultured under identical conditions without tamoxifen. SP600125, etomoxir and puromycin was obtained from MedChem Express.

### Lentiviral infection, knockout and overexpression cells generation

c-Jun knockout cells were generated by CRISPR/Cas9 method. Briefly, recombinant lentivirus delivering single-guided RNAs (5′-GGCGGCGCAGCCGGTCAACG-3′) in lentiCRISPR-V2 vector (Addgene) were produced in HEK293T cells and the lentivirus were used to infect MCF7 and T47D cells, and then selected by puromycin to get puromycin-resistant stable cell line.

For c-Jun overexpression cells generation, the coding sequence of c-Jun with Flag-tag on N-terminal were cloned into pCDH-CMV-MCS-EF1-Puro vector. And the recombinant lentivirus was produced and infected in MCF7 and T47D cell, and get puromycin-resistant cells as c-Jun overexpressed stable cell lines. As the same procedure, the empty-vector infected cells were set as control cell line.

### Cell viability and colony formation assays

Cell viability was assessed by Cell Counting Kit-8 (Dojindo) assay. Cells were seeded at 5000 cells/well into 96-well plates with 100 μl culture medium. The 10 μl CCK-8 solution was added to the cells at specific time points and incubated at 37 °C for 2 h. The reaction product was quantified with the absorbance of 450 nm using Synergy 2 microplate reader (Biotek).

For colony formation assays, cells in single-cell suspension were plated and grown in 6-well plates at a density of 10,000 cells/well followed by tamoxifen or vehicle treatment for 7 days. Later, the colonies were fixed with 4% paraformaldehyde and stained with 0.1% crystal violet.

### RNA extraction and real-time quantitative PCR (RT-qPCR)

Total RNA was extracted from cells using TRI Reagent (Sigma-Aldrich), and 1 μg of total RNA was reverse transcribed using 1st Strand cDNA Synthesis SuperMix (Yeasen). RT-qPCR was performed in triplicates using the Applied Biosystem ViiA TM 7 Real-Time PCR system (Applied Biosystem). The Ct values obtained from different samples were compared using the 2^−ΔΔCt^ method, and the ACTB served as internal reference gene. The primers used for RT-qPCR as follows: 5′-CCCATTCGTAGCCTTTGGTA-3′ (forward) and 5′-AAAACTTGCCCATGTCCTTG-3′ (reverse) for CPT1A; 5′-CTCCTTTCCTTGCTGAGGTG-3′ (forward) and 5′-TCTCGCCTGCAATCATGTAG-3′ (reverse) for CPT1B; 5′-GACCTTCCAGACCAGATCCA-3′ (forward) and 5′-TTGCCAAATAGGGAGAATGG-3′ (reverse) for CPT1C; and 5′-CATGTACGTTGCTATCCAGGC-3′ (forward) and 5′-CTCCTTAATGTCACGCACGAT-3′ (reverse) for ACTB.

### Western blot

Cell lysates were prepared in radioimmunoprecipitation lysis buffer containing 0.1% sodium dodecyl sulfate (SDS), and quantified with the Micro BCA Protein Assay Kit (Thermo Fisher Scientific). 20 μg of protein was electrophoresed trough 10% SDS polyacrylamide gels and were then transferred to polyvinyl difluoride membranes (Millipore). The membranes were blocked with 5% skim milk at room temperature for 1 h and then incubation with primary antibodies at 4 °C overnight. Secondary antibodies were labelled with horseradish peroxidase and the signals were detected using the ECL Kit (Millipore). The β-actin was used as internal control for the whole-cell lysates. Antibody against CPT1A (ab128568, dilution 1:1000), CPT1B (ab134135, dilution 1:1000), c-Jun (ab32137, dilution 1:1000), pS63-c-Jun (ab32385, dilution 1:1000) and JNK1 (ab199380, dilution 1:2000) were purchased from Abcam, CPT1C (12969-1-AP, dilution 1:500) was purchased from Proteintech, CBP (#7389, dilution 1:1000), P300 (#54062, dilution 1:1000), H3 (#4499, dilution 1:2000) were purchased from Cell Signaling Tech, β-actin (A1978, dilution 1:10,000) and Flag (SAB1306078, dilution 1:10,000) were purchased from Sigma-Aldrich.

### CPT1 enzymatic activity

CPT1 enzymatic activity was detected by measuring the release of CoA-SH from palmitoyl-CoA using the general thiol reagent of 5,5′-dithio-bis-(2-nitrobenzoic acid) (DTNB, Sigma-Aldrich). Cell whole lysates were prepared as described for western blot. Whole protein samples and 1 mM DTNB were mixed in the reaction buffer (20 mM Tirs, pH 8.0, 1 mM EDTA) and incubated at room temperature for 30 min. Then 100 μM palmitoyl-CoA (Sigma-Aldrich) and 5 mM L-carnitine (Sigma-Aldrich) were added to the mixture and incubated at 37 °C. As a negative control, reaction buffer was used instead of palmitoyl-CoA. Absorbance was recorded at 412 nm at 1 min intervals for 90 min. CPT1 enzymatic activity was defined as μmol CoA-SH released/min/mg protein.

### FAO rate assessment

Cells were harvest and the mitochondria were extracted using the Cell Mitochondria Isolation Kit (Beyotime). Then the mitochondria were used to assess the fatty acid β-oxidation rate according to the manufacturer’s protocol of the mtCheck™ Fatty Acid β-Oxidation Rate Assay Kit (Creative Biogene). The extracted mitochondria were lysed and the β-oxidation rate was determined by measuring the reduction of palmitoyl carnitine oxidation-dependent ferricyanide.

### ATP level detection

The cellular ATP levels were detected by using a firefly luciferase-based ATP Assay Kit (Beyotime) following the manufacturer’s introduction. Briefly, cells were collected and lysed with lysis buffer and high-speed centrifuged. The precipitation was collected for protein quantification, and the supernatant was collected for ATP detection. 20 μl of each supernatant was added to 100 μl ATP detection working solution and the luminance was measured at Synergy 2 microplate reader (Biotek). The ATP level was defined as the ratio of ATP value (nmol) to protein amount (mg).

### Luciferase assay

HEK293T cells were co-transfected with 500 ng of the luciferase reported plasmid (pGL3-Basic vector based constructs containing 1000 bp sequence upstream of the TSS of CPT1A), 50 ng of the pRL-TK-Renilla-luciferase, and 500 ng of the indicated overexpression plasmids. 24 h after transfection, firefly and Renilla luciferase activities were quantified using the Dual-Luciferase Reporter Assay System (Promega). The CPT1A promoter activity was calculated by the ratio of firefly to Renilla.

### Immunoprecipitation

For the immunoprecipitation assay, cells were lysed in IP lysis buffer containing protease inhibitor cocktail (Sigma-Aldrich) without SDS. The lysates were collected and immunoprecipitated with 1 μg c-Jun antibody (ab32137, Abcam) or IgG antibody at 4 °C overnight followed by incubated with Protein A agarose (Sigma-Aldrich) at 4 °C for 2 h. After that, the immunocomplexes were subsequently washed with IP lysis buffer and subjected to western blot.

### ChIP-qPCR

ChIP assay was performed as described previously [64]. Chromatin was immunoprecipitated with primary antibody of c-Jun (#ab32137, Abcam), CBP (#7389, Cell Signaling Tech), P300 (#54062, Cell Signaling Tech) and H3K27ac (#8173, Cell Signaling Tech), or IgG (#2729, Cell Signaling Tech) overnight at 4 °C. Antibody/chromatin complexes were recovered with Protein G agarose for 2 h at 4 °C, and finally eluted and purified. The immunoprecipitated DNA were quantified by qPCR method. The primers used for ChIP-qPCR as follows: 5′-TGGACACCCATTCTTGAGGT-3′ (forward) and 5′-CCCACGACAGCCCTAGACT-3′ (reverse) for CPT1A.

### Immunofluorescence

Cells were seeded in 6-well plates containing autoclaved glass coverslips. After fixed with ice-cold 100% methanol at −20 °C for 10 min, cells were blocked in Blocking Buffer (5% normal serum, 0.3% Triton X-100 in PBS) at room temperature for 1 h. Then incubating with primary antibody at 4 °C overnight, and incubated in fluorochrome-conjugated secondary antibody diluted in Antibody Dilution Buffer (1% BSA, 0.3% Triton X-100 in PBS) at room temperature in dark for 1 h. Nuclei were counterstained with 4,6-diamidino-2-phenylindole (DAPI). Confocal laser scanning microscopy was performed using Leica TCS SP5 confocal microscope (Leica). Antibody against CPT1A (ab128568) and pS63-c-Jun (ab32385) were purchased from Abcam. The mean fluorescence intensity of the indicated signals in cytoplasm and nucleus were quantified by Image J software.

### Tumour growth studies

Congenitally athymic female nude mice aged 4–6 weeks were housed in laminar flow cabinets under specific pathogen-free conditions with food and water provided ad libitum. We subcutaneously implanted 5 × 10^6^ MCF7-TamR cells in the right axilla of nude mice. Starting day 7 post-implantation, the mice were treated daily with vehicle control, Tam (2 mg/kg in corn oil, by oral gavage), SP600125 (5 mg/kg in 2% DSMO, 2% PEG600 and 2% Tween 80 in PBS, by intraperitoneal injection), or combinations (*n* = 6/group). Mice were monitored for a period of 30 days and were euthanised, and the tumour weight was assessed for all conditions. All procedures were approved by the Animal Ethics Committee of Fujian Medical University.

### Immunohistochemistry (IHC) analysis

IHC staining was performed as described as we described previously [65]. The paraffin sections of 5 μm thickness were prepared from tissue microarrays. The sections were deparaffinised, treated with 3% H_2_O_2_, and autoclaved in 10 mM sodium citric (pH 6.0) for heat-induced antigen retrieval, and then incubated with primary antibodies at 4 °C overnight, followed by incubation with biotinylated secondary antibody at room temperature for 1 h. Finally, 3,3-diaminobenzidine tetrahydrochloride was used as colouring reagent, and haematoxylin was used as a counterstain for nuclei. The stained sections were photographed at a light microscope equipped with a camera (Olympus). Antibody against CPT1A (ab128568) and pS63-c-Jun (ab32385) were purchased from Abcam.

The quantification of IHC staining was based upon the staining intensity and the percentage of positive stained cells. The intensity was recorded as 0, 1, 2, and 3, referring to negative, weak, moderate, and strong staining, respectively. The percentage of positive stained cells was recorded from 0 to 100%. The results of staining were scored using the Histoscore, which was obtained by multiplying the percentage of positive cells by the intensity. The median value of the Histoscore (150) were used as cutoff points to classify protein as low or high expression.

### Bioinformatics analysis

The date set of GSE144378 [19] was downloaded from the public source GEO data repository (http://www.ncbi.nlm.nih.gov/geo/). The enrichment analysis based on the Gene Ontology processes was performed using the web-based platform Metascape [66]. The transcription factor enrichment analysis for the co-upregulated genes was performed by ChIP-X Enrichment analysis 3 (ChEA3) web server [24].

### Statistical analysis

All studies were performed at least three independent experiments, and data presented as mean ± SEM from three biological replicates. Group comparison of normally distributed measurement data and categorical data were performed using unpaired or paired Student’s *t* text and Chi-square test, respectively. The Kaplan–Meier survival plot, HR and log-rank *P* value were used to analysis the prognostic factors of ER-positive BC patients. Pearson correlation coefficient were used to analysed the correlation between CPT1A and pS63-c-Jun protein expression in tissue microarray, or tamoxifen sensitivity and c-Jun mRNA expression obtained from Cancer Dependency Map. The differences were considered statistically significant if *P* < 0.05, and indicated by *P < 0.05, **P < 0.01, ***P < 0.001; or ns, not significant.

### Supplementary information

Supplementary materials

Original Data File

Reproducibility checklist

## Data Availability

The authors declare that there are no primary datasets and computer codes associated with this study. The datasets used and/or analysed during the current study are available from the corresponding author on reasonable request.
