# Supplementary material for: Targeting c-Jun inhibits fatty acid oxidation to overcome tamoxifen resistance in estrogen receptor-positive breast cancer
Source: Cell Death Dis. 2023 Oct 6;14(10):653. doi: 10.1038/s41419-023-06181-5 (PMC10558541; doi:10.1038/s41419-023-06181-5)
Supplement: Supplementary file 1 — Supplementary materials [file 41419_2023_6181_MOESM1_ESM.pdf]

Supplementary material for

Targeting c-Jun inhibits fatty acid oxidation to overcome tamoxifen resistance in estrogen receptor-positive breast cancer

Cen Jiang, et al.

Contents:

7 Supplementary Figures and Legends

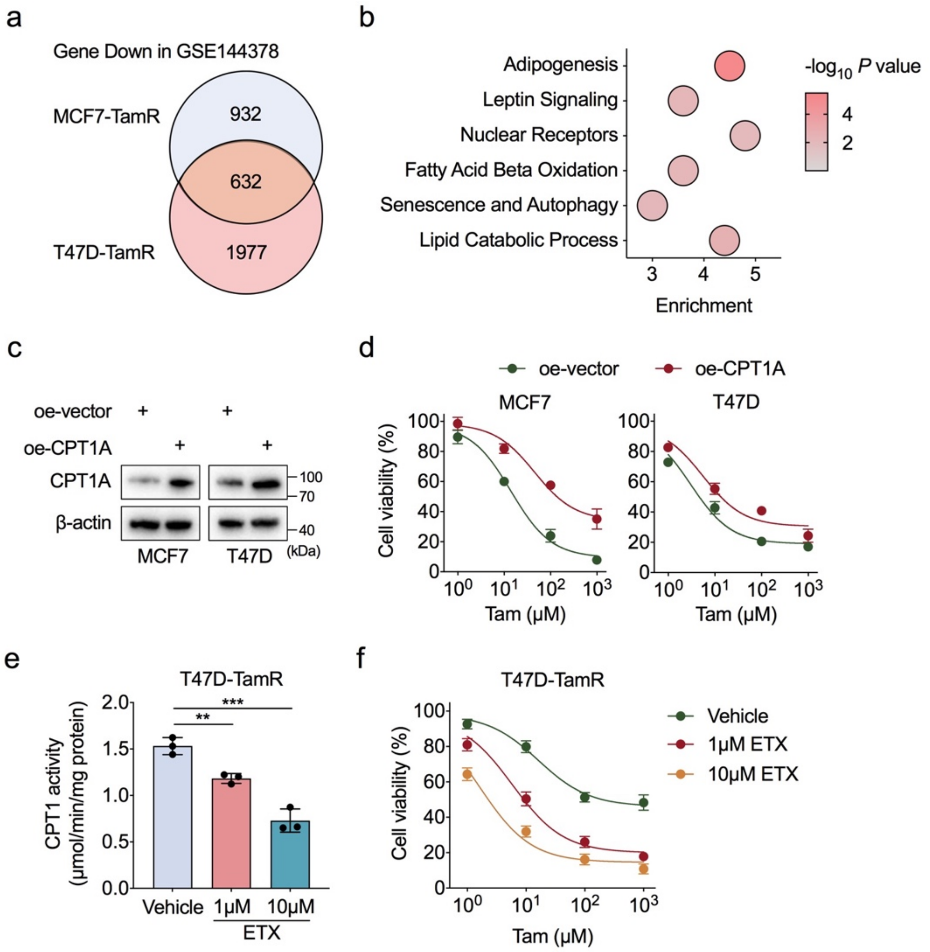

Supplementary Fig. 1. FAO confers tamoxifen resistance in ER-positive BC cell, Related to Figure 1. (**a**) Venn diagram of overlapping genes significantly downregulated in tamoxifen-resistant (TamR) MCF7 and T47D cells identified in the GEO dataset of GSE144378. (**b**) GO analysis of the significantly enriched biological processes for 632 downregulated genes in both MCF7-TamR and T47D-TamR cells. (**c**). Western blot analysis to validate the CPT1A overexpression efficiency in wild-type MCF7 and T47D cells. (**d**). CCK-8 analysis of wild-type MCF7 and T47D cells transfected with CPT1A-overexpressing plasmids or empty vector treated with a concentration gradient of tamoxifen for 72 h. (**e**) Comparison of CPT1 enzymatic activities in T47D-TamR cells with or without ETX treatment for 24 h. Unpaired student's *t* test; $^{**}P<0.01$, $^{***}P<0.001$. (**f**) CCK-8 analysis for T47D-TamR cells treated with tamoxifen combined with or without ETX for 72 h.

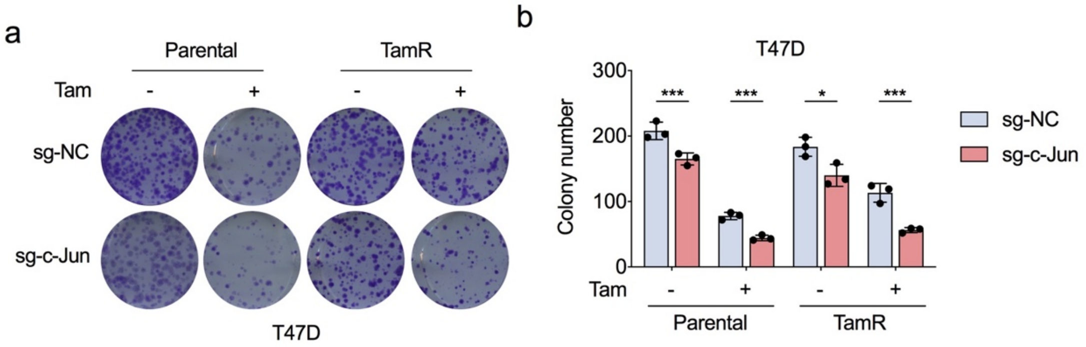

Supplementary Fig. 2. c-Jun deletion restores tamoxifen sensitivity, Related to Figure 2. (**a**) Representative images of colony formation assays for TamR and parental T47D cells with or without c-Jun knockout treated with tamoxifen (10 μM) or vehicle and stained with crystal violet. (**b**) Quantification results of the colony formation assays in (**a**). Unpaired student's t test; $^{*}P<0.05$, $^{***}P<0.001$.

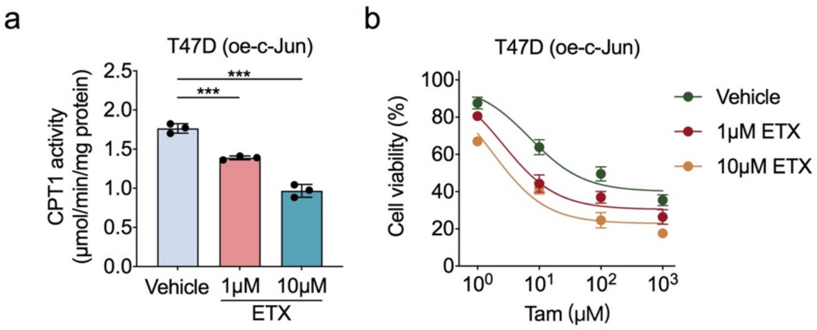

Supplementary Fig. 3. c-Jun activates FAO to induce tamoxifen resistance, Related to Figure 3. (**a**) CPT1 enzymatic activities in c-Jun overexpressed wild-type T47D cells with or without ETX treatment for 24 h. Unpaired student's *t* test; $^{***}P<0.001$. (**b**) CCK-8 analysis for c-Jun overexpressed wild-type T47D cells treated with concentration-gradient of tamoxifen combined with or without ETX for 72 h.

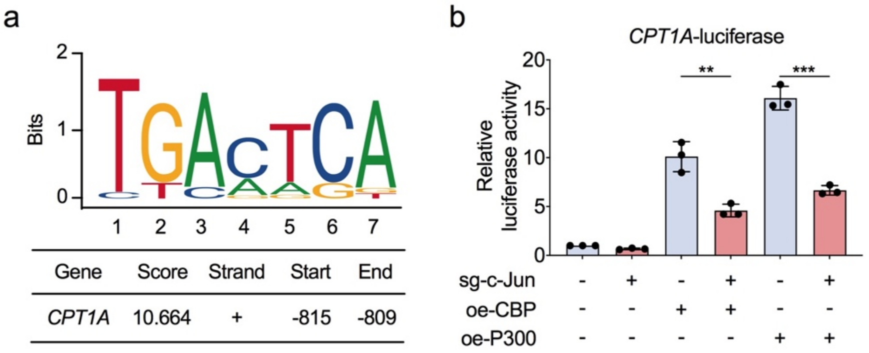

Supplementary Fig. 4. c-Jun recruits the CBP/P300 to activate CPT1A transcription, Related to Figure 4. (**a**) The predicted c-Jun binding site (TRE motif) on the promoter of CPT1A, analyzed by the JASPAR database. (**b**) HEK293T cells were transfected with c-Jun-knockout construct, CBP- or P300-overexpressed plasmid alone or together as indicated to analyze the luciferase reporter activity driven by CPT1A promoter. Paired Student's *t* test; $^{**}P<0.01$, $^{***}P<0.001$.

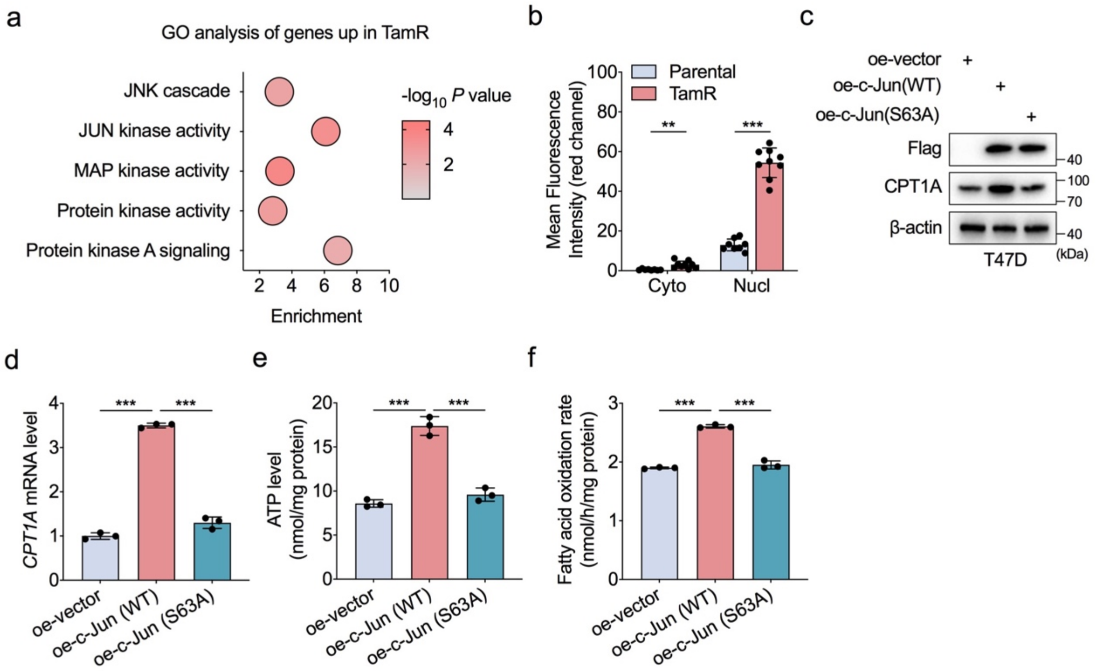

Supplementary Fig. 5. JNK-dependent c-Jun phosphorylation activates FAO, Related to Figure 5. (**a**) GO analysis of the significant enriched kinase related biological processes for 334 up-regulated genes in MCF7-TamR and T47D-TamR cells. (**b**) Statistical analysis for the mean fluorescence intensity of the pS63-c-Jun signals in the cytoplasm and nuclei between TamR and parental T47D cells in Fig. 5a. (**c-f**) Comparison of CPT1A protein levels (**c**), CPT1A mRNA levels (**d**), cellular ATP levels (**e**), and FAO rates (**f**) in T47D cells transfected with empty vector, wild-type c-Jun construct (WT), or Ser63 phosphorylation disabled mutant form of c-Jun construct (S63A). Unpaired Student's *t* test in **b**, **d**, **e**, and **f**; $^{**}P<0.01$, $^{***}P<0.001$.

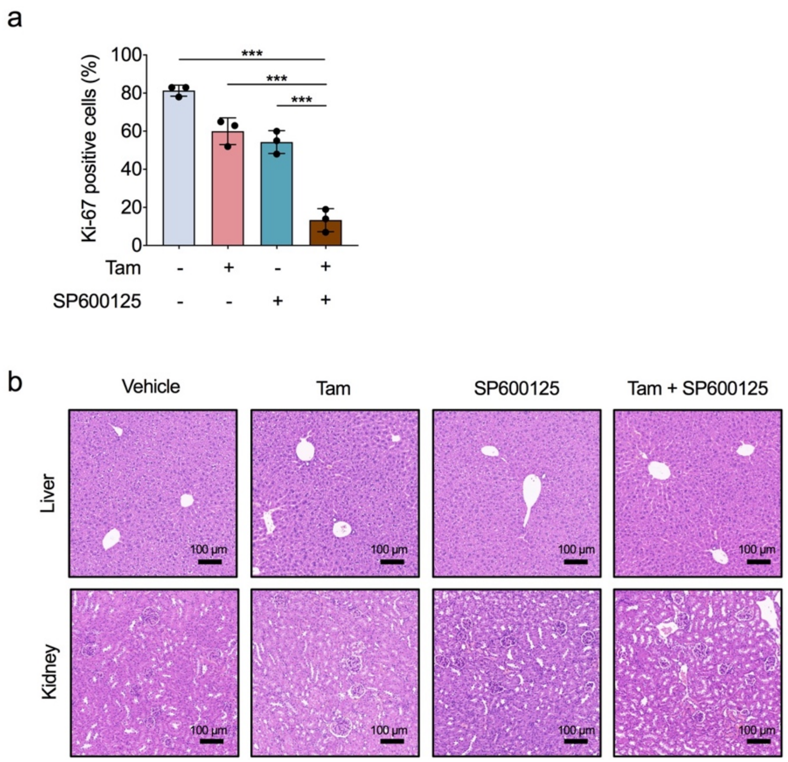

Supplementary Fig. 6 JNK inhibitors sensitize ER-positive BC cells to tamoxifen by inhibiting c-Jun-induced FAO, Related to Figure 6. (**a**) Ki-67 staining quantitation results for Fig. 6h. Unpaired Student's *t* test; $^{***}P<0.001$. (**b**) H&E stainings were used to confirmed neither hepatotoxicity nor nephrotoxicity in mice undergoing the indicated treatments.

a

		pS63-c-Jun high	pS63-c-Jun low	X^2	*P*
CPT1A	high	34	10	21.177	<0.001
	low	13	35		

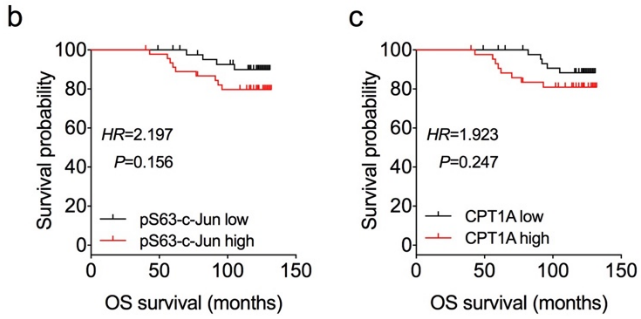

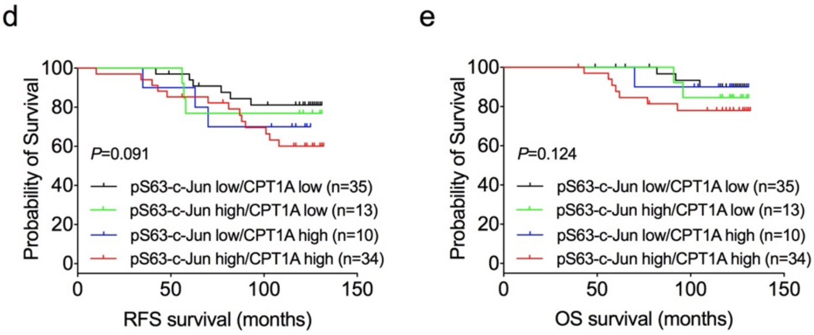

Supplementary Fig. 7. c-Jun predicts tamoxifen therapy outcomes in ER-positive BC patients, Related to Figure 7. (**a**) Statistical analysis was conducted based on the protein level of pS63-c-Jun and CPT1A in ER-positive BC tissues. Chi-square test. (**b**) Overall survival (OS) was compared between patients with high and low expression of pS63-c-Jun. (**c**) OS was compared between patients with high and low expression of CPT1A. (**d**) Recurrence-free survival (RFS) were compared between patients with different expression status in combination of pS63-c-Jun with CPT1A. (**e**) OS were compared between patients with different expression status in combination of pS63-c-Jun with CPT1A. Log-rank test in **b**, **c**, **d**, and **e**.
