# Supplementary figures and images for: Targeting c-Jun inhibits fatty acid oxidation to overcome tamoxifen resistance in estrogen receptor-positive breast cancer
Source: Cell Death Dis. 2023 Oct 6;14(10):653. doi: 10.1038/s41419-023-06181-5 (PMC10558541; doi:10.1038/s41419-023-06181-5)

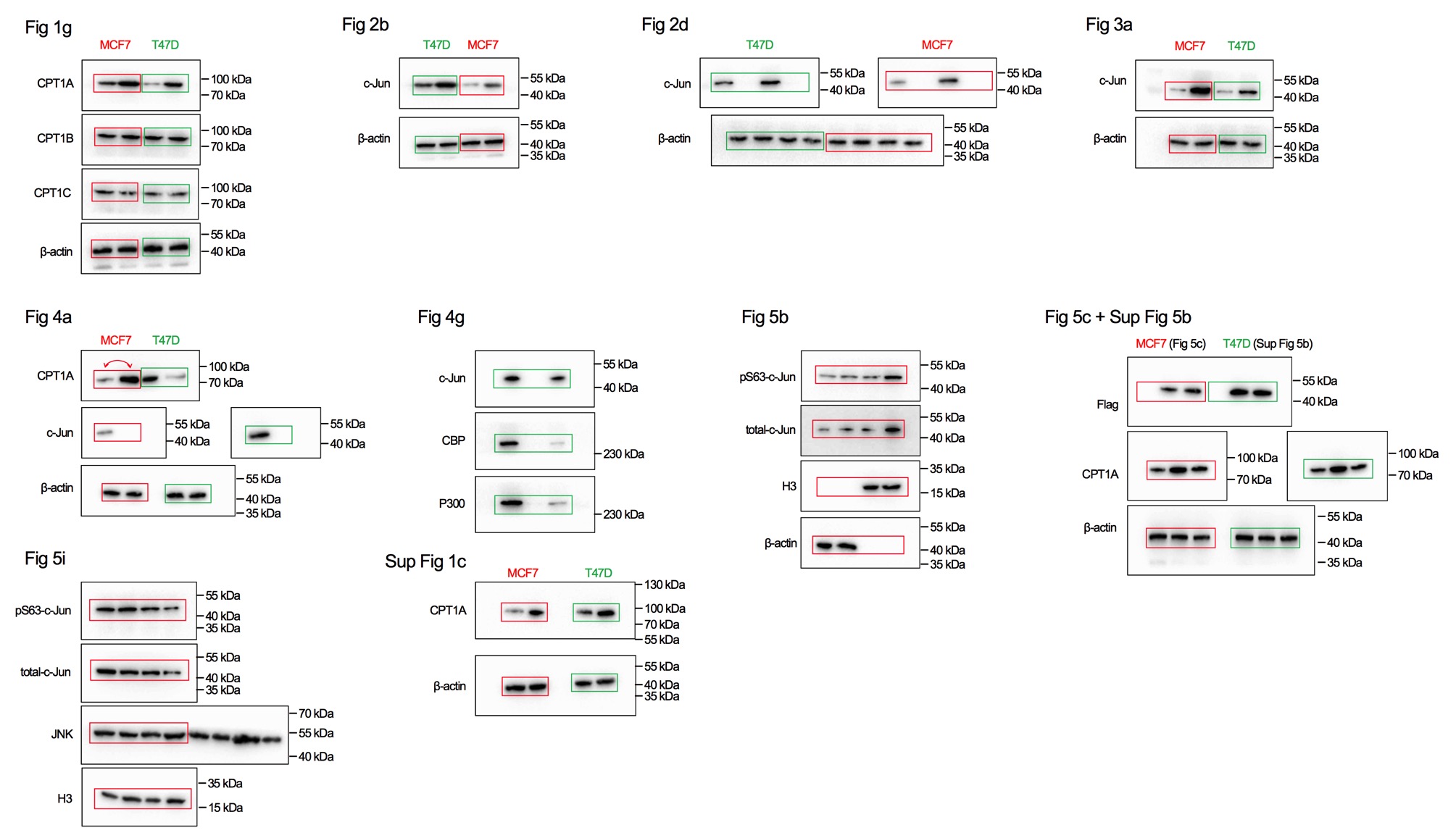

Supplement: Supplementary file 2 — Original Data File [file 41419_2023_6181_MOESM2_ESM.jpg]
